# Modified Pechini Processing of Barium and Lanthanum-Lithium Titanate Nanoparticles and Thin Films

**DOI:** 10.1186/s11671-017-2123-8

**Published:** 2017-05-12

**Authors:** A. Suslov, S. Kobylianska, D. Durilin, O. Ovchar, V. Trachevskii, B. Jancar, A. Belous

**Affiliations:** 1 V.I. Vernadskii Institute of General and Inorganic Chemistry of N.A.S of Ukraine, 32/34 Palladina Ave., 03680 Kyiv, Ukraine; 2 G. V. Kurdyumov Institute for Metal Physics of the N.A.S. of Ukraine, Kyiv, Ukraine; 3 Jozef Stefan Institute, Jamova 39, 1000, Ljubljana, Slovenia

**Keywords:** Perovskite structure, Thin films, Pechini method

## Abstract

Barium-strontium titanate (BST) Ba_0.6_Sr_*0.4*_TiO_3_ and lanthanum-lithium titanate (LLT) La_0.5_Li_0.5_TiO_3_ nanopowders and thin films have been obtained via the modified Pechini route. Polyesterification and complexation processes of gel formation have been examined. Hypothetical models of coordinative polymers formed in sol-gel system have been suggested. It has been shown that BST and LLT solid solutions form in one step at relatively low temperature. X-ray diffraction confirms that the final products, which are single phases and have cubic shape, are formed at 600 and 700 °C for BST and LLT respectively. It has been found that use of thermal shock as pretreatment allows to increase the density of BST- and LLT-based thin films.

## Background

Titanates of alkaline, alkaline earth, and rare earth elements with perovskite structure are widely used in modern technology and a range of materials with a set of functional properties is based on them. A great attention attract barium-strontium titanate (BST)-based dielectrics [1, 2] and lanthanum-lithium titanate-based ionic conductors [3, 4].

Ba_1−*x*_Sr_*x*_TiO_3_ (BST) solid solutions are promising candidates for applications in electro-optical devices, MEMs, tunable microwave devices, and multilayer capacitors (MLCCs) [5, 6].

Among the ionic conductors, La_0.5_Li_0.5_TiO_3_ (lanthanum-lithium titanate (LLT)) with defect perovskite structure attracts a great interest. A high lithium-ionic conduction of LLT allows to involve them in the development of electrochemical energy source components [4].

The conventional technique for Ba_1−*x*_Sr_*x*_TiO_3_ and La_0.5_Li_0.5_TiO_3_ preparation involves reaction in the solid phase at temperatures around 1000 ± °C [5, 6]. This method however does not allow to achieve single phase and sufficient chemical homogeneity that could impair functional properties of materials [7, 8]. Besides, high calcination temperature leads to uncontrolled particle growth, and therefore, high temperature is required for their sintering [9]. In the case of La_0.5_Li_0.5_TiO_3_, high temperature of sintering results in lithium evaporation and stoichiometry deviation [10]. At the same time, phase and chemical homogeneity of these materials could be improved by using Pechini method [11, 12]. Pechini method allows to produce gels which could be used for producing thin films of materials based of BST and LLT. BST thin films are very promising for microwave tunable applications due to the low tuning voltages and the relatively low production cost [8]. In the case of LLT-based materials, thin film production gives possibilities to develop miniaturized batteries for different technical and medical applications [13, 14].

Pechini method are widely used for synthesis of BST and LLT materials. However, there are only a few research works [15, 16], devoted to the study of chemical processes and phase transformations in the chain: initial reagents–solution system (metal containing polymeric gel)–precursor–final material [11, 17, 18]. Besides, there are not enough information about optimal thin film deposition and annealing conditions to high film quality achievement.

The present work concerns the study of polymerization and complexation processes accompanying BST and LLT sol-gel solution formation, BST and LLT thin film production via the chemical solution deposition method as well as the impact of heat treatment on the thin film microstructure and density.

## Methods

The BaCO_3_ (99.99% Merck), SrCO_3_ (99.999%, Cerac), Li_2_CO_3_ (99.99% Merck), La_2_O_3_ (99.999%, Cerac), diisopropoxytitanium bis(acetylacetonate) solution (~75% in isopropanol) (purum, Sigma-Aldrich), citric acid (CA) (anhydrous, 99%, Merck), ethyleneglycole (EG) (99% Sigma-Aldrich), and acetic (99–100%) and nitric (60%) acids (both Merck) were chosen as started reagents. Citric acid was dissolved in ethylene glycol in 1:4 molar ratios and boiled for 1 h. In the case of BST predetermined stoichiometric amounts of diisopropoxytitanium bis(acetylacetonate) solution and solution of barium, strontium acetates in acetic acid were added to it under vigorous stirring. The barium, strontium, and magnesium acetates were prepared by dissolving weighed amounts of BaCO_3_ and SrCO_3_ in glacial acetic acid. In the case of LLT predetermined stoichiometric amounts of titanium β-dicetonate solution and solutions of lithium and lanthanum nitrates in the nitric acid were added to the polymeric gel. Lanthanum and lithium nitrates were prepared by dissolving of lanthanum oxide and lithium carbonate in nitric acid.

BST and LLT thin films were deposited on the α-Al_2_O_3_ polycrystalline substrates by spin-coating method. The method of spin-coating involves depositing a small puddle of previously prepared sol-gel solution onto the center of a substrate and then spinning the substrate at high speed. Under the influence of centrifugal force, gel evenly cover the surface of the substrate. Application installation was carried out by SCI-20 (Novocontrol Technologies GmbH & Co, KG) with the speed of rotation 3600 rpm. BST and LLT thin films were deposited in three layers with heat treatment between each layer. Heat treatment has been carried out in two ways. In the first case, BST and LLT green films have been dried at 150–200 °C for 30 min. In the second case, thermal shock has been applied: BST and LLT green films have been placed in a furnace preheated to 600 °C for 30 min. This procedure was repeated before each subsequent thin film layer deposition. After pretreatment, films have been sintered in the temperature range 600–1000 °C with heating rate of 5 °C/min and then furnace-cooled.

Phase composition of BST and LLT thin films was investigated by X-ray diffractometer DRON 4M (CuK_α_ radiation, SiO_2_ calibration standard, step-scan mode with a step size of 0.02°, and a counting time per data point of 6 s).

SEM analysis was carried out in a JEOL JXA 840A with magnification ×50,000, ×60,000, ×120,000.

The 13C-NMR spectra were recorded on a Bruker AVANCE 400 instrument. NMR spectra was performed with solutions placed in quartz cuvette (*d* = 1 mm). As external standard, tetrametylsylan ((CH_3_)_4_Si) was used.

Infrared spectra were measured at room temperature on a SPECTRUM 100 PERKIN ELMER spectrometer in the range 400–4000 cm^−1^. Spectra were filmed with a thin layer of solution deposited between two plates of KBr.

## Result and Discussion

### Polymeric Gel Formation

**Fig. 1:**
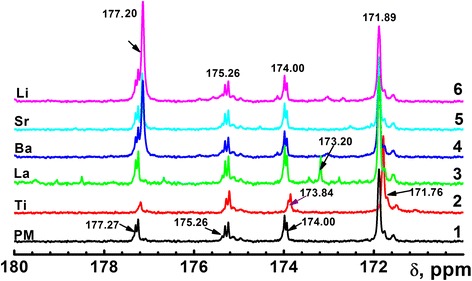
13C-NMR spectra of the polymer matrix (1) and polymer matrix with metal ions: Ti4+ (2), La3+ (3), Ba2+ (4), Sr2+ (5), Li+ (6)

**Fig. 2:**
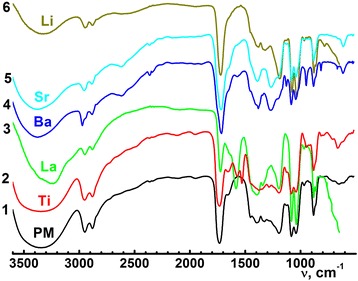
FT-IR-spectra of polymeric matrix (1) and polymeric matrix with metal ions: Ti4+ (2), La3+ (3), Ba2+ (4), Sr4+ (5), Li + (6)

According to the ^13^C-NMR spectra (Fig. 1, curve 3), interaction between citric acid and ethyleneglycol results in formation of the three types of polyesters: the two types of linear polyesters (Fig. 2, products 1 and 2) and branched polyester (Fig. 2, product 3). The existence of such polymers arising from presence of the signals of the central (174.00) and terminal (171.28) carboxyl groups which are not involved in condensation reaction ((Fig. 1, curve 1), signals at 177.27 and 175.26 ppm in the ^13^CNMR spectra, are due to the terminal and central ester groups of the citric polyester, respectively [11]. It is suggested that in formed polymeric gel not involved in polyesterification reaction, carboxyl groups are present. Therefore, a few types of metal (La^3+^ and Ti^4+^) complexes are possible to be formed: with terminal carboxyl groups, central carboxyl groups, and their mixture.

### Polymeric La^3+^ and Ti^4+^ Complexes Formation

In the ^13^C-NMR spectrum of the Ti^4+^ (in both BST and LLT solution systems) polymeric solutions, the shifted peaks from 174.00 to 173.82 ppm (Fig. 1) for the terminal free carboxyl groups indicate the formation of the Ti^4+^ polyester complex, whereas Ti^4+^ ions are coordinative bounded with terminal carboxyl groups. Carboxyl groups coordination was confirmed by FT-IR spectra (Fig. 2). In the FT-IR spectra of the polymeric gel with Ti^4+^ ions, the splitted bands in the region 1605 and 1400 cm^−1^ correspond to stretching vibrations of coordinated carboxyl groups [19]. Given the fact that the typical coordination number of the Ti^4+^ is 6 [20], the coordinative surround of the Ti^4+^ cannot be saturated only with carboxyl groups of the polymeric gel citric fragments because of the steric features of the latests. In our opinion, the most suitable ligand to fill the coordination surroundings is the acetylacetone, which molecules are present in the initial Ti^4+^ acetylacetonate. Therefore, the hypothetical structure of possible polymeric complex fragment of Ti^4+^ is shown in Fig. 2a. In the case of La^3+^ addition into polymer matrix, the appearance of new peak 173.18 ppm in the ^13^C-NMR spectrum has been indicated (Fig. 1, curve 3). These peaks can be attributed to coordination La^3+^ with both carboxyl and ester groups. In the FT-IR spectra, strong band at 1580 cm^−1^ confirms the coordination La^3+^ with carboxyl groups and suggests the high ionicity of this metal-ligand bond [21]. These facts suggest different coordination behavior of the Ti^4+^ and La^3+^ cations [21].

### Ba^2+^, Sr^2+^, and Li^+^ behavior

**Fig. 3:**
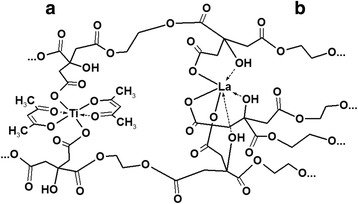
Hypothetical structure of the heterometalic La3+ (**a**) and Ti4+ (**b**)—polymeric complex fragments derived from 13C-NMR and FT-IR spectra

In the ^13^C-NMR spectra (Fig. 1) of the polymeric gel after barium and strontium acetates and lithium nitrate addition, changes of the free carboxyl groups’ positions of the peaks have not been detected. Moreover, any appreciable changes in the IR spectra of the polymeric gel after barium and strontium acetates and lithium nitrate addition have not been observed too. These facts suggest the barium, strontium, and lithium ions not be involved in complex formation (Fig. 3).

Therefore, in the BST and LLT solid solutions by Pechini method preparation, only Ti^4+^ and La^3+^ form polymeric complexes, while the Ba^2+^, Sr^2+^, and Li^+^ are not included in the polymeric complex. However, this fact does not lead to the polymeric gel instability: any precipitation or turbidity of both BST and LLT polymeric gel systems have not been detected.

### BST and LLT in Thin Film Form Formation

**Fig. 4:**
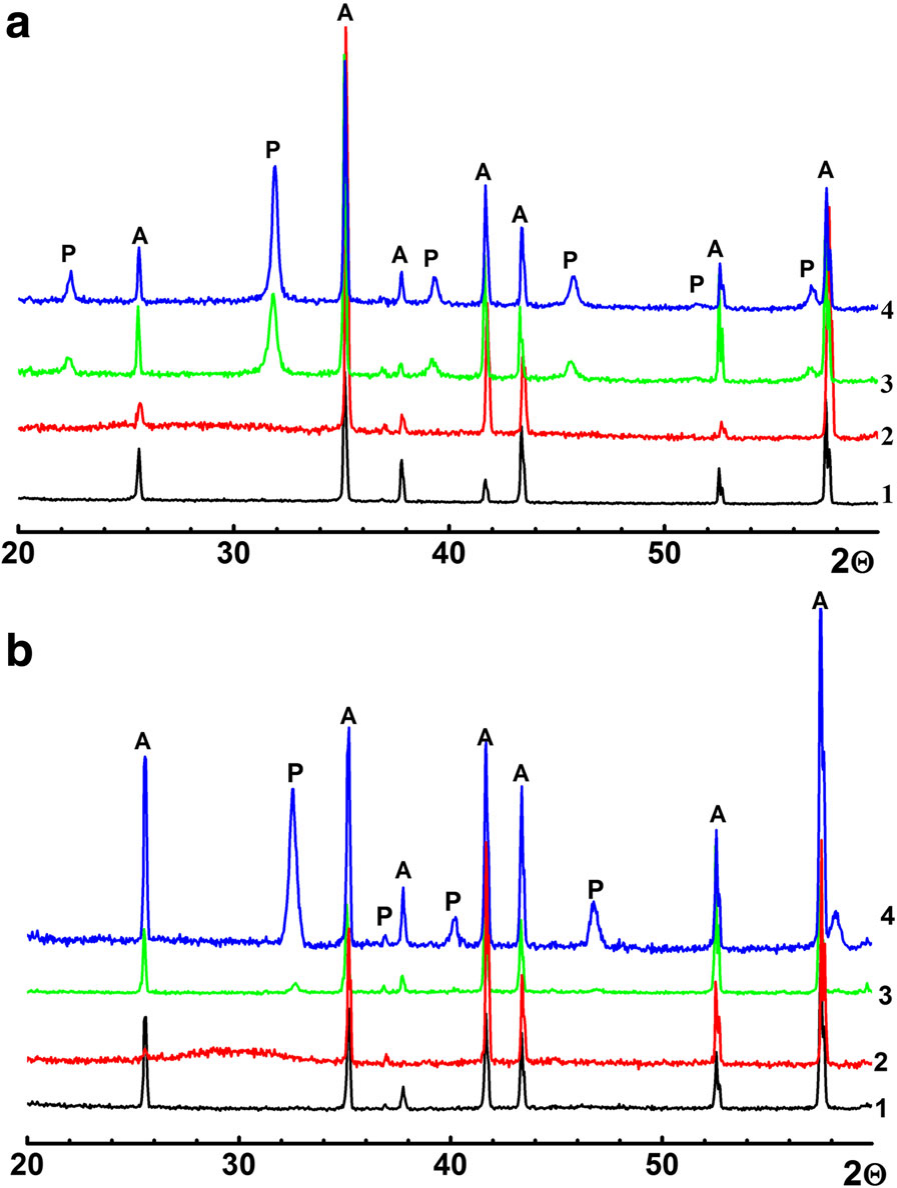
X-ray diffraction patterns of α-Al2O3 substrate (1) and the thin films after heat treatment at different temperatures. **a** BST: 2—500, 3—600, and 4—900 °C. **b** LLT: 2—500, 3—700, and 4—900 °C

X-ray diffraction patterns of BST and LLT thin films, obtained in the temperature range 500–700 °C respectively, are showed in Fig. 4. According to these data, there have not been found secondary phases in sintered films. Moreover, any intermediate phases during sintering in the all temperature range have not been detected. Therefore, both of BST and LLT perovskite phase formation occurs in one step. Eventually, this fact contributes to the BST and LLT thin films with high chemical and phase homogeneity achievement. Obtained thin films can be satisfy described as a cubic with lattice parameter *a* = 3.965 Å and tetragonal with lattice parameters *a* = 5.482 Å and *c* = 7.746 Å for BST and LLT respectively.

### Heat Treatment Effect on the BST and LLT Thin Film Microstructure

The heat treatment of films are much more complicated than of ceramic with the same composition. The green tape of thin films contain more than 95% organic compounds (polymeric complexes and EG as a solvent). The heat treatment of thick films should be carried out in several stages, where the first stage is necessary to remove organic components (annealing or pretreatment), and the second—for sintering grains (sintering of films). The first stage is particularly important. Annealing of the films can be realized by two ways: slow heating at 150–200 °C or fast heating at 600 °C (thermal shock). Sintering of films were made by slow heating rate (60 °C/h) till 800–1000 °C.

**Fig. 5:**
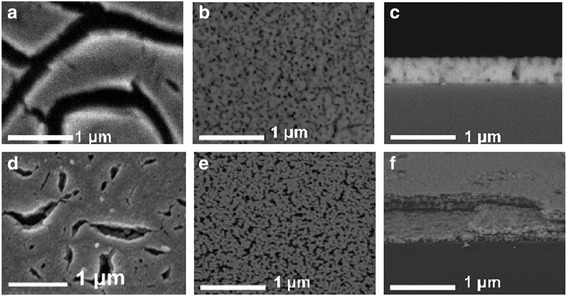
SEM-images of BST (**a**–**c**) and LLT (**d**–**f**) thin film surface (**a**, **b**, **d**, **e**) and cross sections (**c**, **f**), where thin films a (**c**) and d (**f**) have been obtained via slow drying and with thermal shock usage (**b**, **c**, **e**, **f**)

According to microstructural studies, a significant impact on the microstructure heat treatment regimes were found. In the case of dying at 150–200 °C, a great number of different defects such as porous, cracks, and exfoliation from the substrate for BST and LLT thin films have been observed (Fig. 5a, d). These defect appearance can be attributed to the deformations of the polymer component framework—citric polyester—as a result of light boiling components of gel (ethylene glycol, acetylacetone, and acetic acid) evaporation and polyester concentration.

Other way was a rapid heating (thermal shock) at 500–600 °C for pyrolysis of organic components. It has been shown that applying thermal shock allows to improve the BST and LLT film microstructure significantly (Fig. 5b, e). Microstructure quality enhancing via thermal shock can be explained as a result of thin film structure fixation which related with the fast organic compound destruction. The thickness of BST and LLT thin films were of 450–500 and 700–750 nm respectively (Fig. 5c).

## Conclusions

Polyesterification and complexation processes accompanying BST and LLT sol-gel solution formation have been studied. It has been shown that only Ti^4+^ and La^3+^ take part in polymeric complexes formation. Ba^2+^, Sr^2+^, and Li^+^ are not included in polymeric complexes. Ti^4+^ and La^3+^ polymeric complexes hypothetical structure have been suggested. BST and LLT phases in thin film form have been shown to form in one step at relatively low temperatures: 600 and 700 °C for BST and LLT respectively. It have been found that use of thermal shock in both of BST and LLT thin film production allows to reduce the appearance of pores and cracks significantly.

## Abbreviations

BST: Barium-strontium titanate
CA: Citric acid
EG: Ethyleneglycole
LLT: Lanthanum-lithium titanate
